# Development of COVID-19 severity assessment score in adults presenting with COVID-19 to the emergency department

**DOI:** 10.1186/s12879-022-07535-8

**Published:** 2022-06-27

**Authors:** Faysal Subhani, Abdul Ahad Chhotani, Shahan Waheed, Rana Osama Zahid, Kiran Azizi, Ahmed Raheem Buksh

**Affiliations:** Department of Emergency Medicine, Aga Khan University Hospital, Stadium Road, Karachi, 74800 Pakistan

**Keywords:** Covid-19, Prediction score, qSOFA, Emergency

## Abstract

**Background:**

Critically-ill Covid-19 patients require extensive resources which can overburden a healthcare system already under strain due to a pandemic. A good disease severity prediction score can help allocate resources to where they are needed most.

**Objectives:**

We developed a Covid-19 Severity Assessment Score (CoSAS) to predict those patients likely to suffer from mortalities within 28 days of hospital admission. We also compared this score to Quick Sequential Organ Failure Assessment (qSOFA) in adults.

**Methods:**

CoSAS includes the following 10 components: Age, gender, Clinical Frailty Score, number of comorbidities, Ferritin level, D-dimer level, neutrophil/lymphocyte ratio, C-reactive Protein levels, systolic blood pressure and oxygen saturation. Our study was a single center study with data collected via chart review and phone calls. 309 patients were included in the study.

**Results:**

CoSAS proved to be a good score to predict Covid-19 mortality with an Area under the Curve (AUC) of 0.78. It also proved better than qSOFA (AUC of 0.70). More studies are needed to externally validate CoSAS.

**Conclusion:**

CoSAS is an accurate score to predict Covid-19 mortality in the Pakistani population.

## Introduction

The COVID-19 pandemic has challenged healthcare systems of both developed and developing countries. Literature shows that the elderly and those with comorbid conditions are highly vulnerable. These critically ill COVID -19 patients require expensive resources such as intensive care and ventilatory support. This overburdens already-fragile healthcare systems in many low-resource healthcare systems like Pakistan. Therefore, judicious allocation of resources is imperative but often challenging.

Prognosticating a rapidly spreading pandemic is difficult due to a rapid influx of data with weak study methods and a multitude of factors that predict poor outcomes [1]. Numerous scores have been developed to predict poor outcomes. However, there is generally a lack of consensus among emergency and critical care physicians in applying such scores in practice [2]. The Quick Sequential Organ Failure Assessment (qSOFA) is a score that is widely accepted and used as a tool in predicting severity of disease in emergency departments (EDs). The usefulness of qSOFA in low- and middle-income countries has not been well established and further prospective validation in low- and middle-income settings is needed [3]. Furthermore, the utility of this score in COVID-19 is not well established. Other scores developed include the VACO Index [4], 4C mortality score [5] and COVID-GRAM [6]. Additionally, scores previously used for non-Covid pneumonia such as CURB-65 have also been tested on COVID-19 patients [7]. These scores accurately predict severe disease in COVID-19 patients. However, they often suffer from certain deficiencies, limiting their generalized applicability. For example the VACO index, an otherwise thoroughly investigated and validated score that demonstrates that age is a major driver of COVID-19 mortality, has significantly different Area under the Curves for different subgroups, implying limited generalizability of the score in various populations. Having said that, it still maintains good discrimination for 30 day mortality within these various subgroups [4]. Another score, the COVID-GRAM, has good predictive ability in low-risk patients for progression to severe disease, but overestimates risk in high-risk patients [6].

**C**ovid-19 **S**everity **A**ssessment **S**core **(CoSAS)** is a tool that was developed to allow emergency physicians and intensivists to identify patients more likely to die from COVID-19. The tool is expected to further aid in deciding ceiling of care beforehand and subsequently conserve resources in the current pandemic. The variables of CoSAS were selected based on their early availability, easy measurement in EDs in Pakistan and were ideal due to the following reasons: (1) they are all objective and can be measured easily, (2) the selected laboratory parameters are believed to predict severity of disease based on their role in disease pathophysiology and (3) previous studies have shown their validity [1, 8, 9]. The selected predictors will be evaluated through a prospective study design and subsequently internally validated through a collected data set. Therefore, in the present study we aim to validate the COVID-19 Severity Assessment Score (CoSAS) and compare it with qSOFA in predicting outcomes.

## Materials and methods

### Study setting, design and sample size calculation

This is a hospital based observational study undertaken in the department of Emergency Medicine of Aga Khan University Hospital over a period of six months from March 2020 to July 2020, which is a 550 bedded large tertiary care teaching facility located in Karachi, Pakistan. All adults > 18 years presenting to the emergency department and admitted to the Intensive Care Unit or Special Care Unit with COVID-19 (RT-PCR test positive) at any point in their hospital stay were included. Patients needing admission in general ward beds were excluded. This was done to allow for the fact that the elderly are more likely to come to the hospital before the young. Excluding those needing admission to the general ward allowed us to include only those who were quite sick at the time they presented to the emergency department.

Liu et al. [10] reported an AUC of 0.74 on ROC curve analysis in predicting mortality using qSOFA score. With a 95% confidence interval, expected area under the curve (AUC) of 0.89, and 3% margin of error, a minimum required sample size of n = 145 was calculated. Sample size for the study based on the area under the curve (AUC) was calculated using the method defined by Hajian-Tilaki et al. [11] using Microsoft Excel. Formula used for the sample size calculation was as below;

$$n=\frac{{Z}_{\left(\frac{\alpha }{2}\right)}^{2}V(\widehat{AUC})}{{e}^{2}}$$

where, $$V(AUC)=\left(0.0099{e}^{-\frac{{\varnothing }^{2}}{2}}\right)(6{\varnothing }^{2}+16)$$ and $$\varnothing =1.414{Z}_{AUC}$$

There are two main variables in this study that are essentially the scoring models, namely qSOFA and CoSAS. The qSOFA and CoSAS were calculated using data collected within 24 h of ED presentation. CoSAS is a severity assessment score that contains different variables given in Table 1. We defined a cutoff value of ≥ 6 for severe Covid-19 pneumonia. qSOFA variables are listed in Table 2. Two or more positive variables indicate a poor prognosis in the score. Each variable in both scores can either score 1 or 0 points (as defined in Tables 1 and 2). Maximum CoSAS score is 10 and maximum qSOFA score is 3. A higher qSOFA score is associated with worse outcomes and we posit that a higher CoSAS score would do the same.

**Table 1:** CoSAS variables

Serial Number	Features	Scoring
1	Age ≥ 50 years at the time of scoring [12]	≥ 50 = 1, < 50 = 0
2	Male Gender [12]	Male = 1, Female = 0
3	2 or more Comorbid (Diabetes, Hypertension, Cardiovascular disease (cardiomyopathy, chronic heart failure, Ischemic heart disease), Kidney Disease (nephrotic and nephritic syndrome, chronic kidney disease), Respiratory disease (obstructive and restrictive lung diseases), Cerebrovascular Disease, Immunosuppressive disease (malignancy, HIV, any disease process requiring long-term immunosuppressive therapy)) [13]	≥ 2 = 1, < 2 = 0
4	Clinical Frailty Scale (≥ 7) [14]	≥ 7 = 1, < 7 = 0
5	Oxygen Saturation < 92% on room air (at presentation/clinical deterioration)	< 92% = 1, ≥ 92% = 0
6	Systolic Blood Pressure ≤ 100 mmHg	SBP ≤ 100 = 1, SBP > 100 = 0
7	Neutrophil/Lymphocyte Ratio (NLR) (9–18) [15]	NLR > 18 = 1, NLR ≤ 18 = 0
8	C-reactive protein raised (CRP) [15]	CRP > 10 mg/L = 1, CRP ≤ 10 mg/L = 0
9	D-Dimer (DID) raised [16]	DID > 0.5 = 1, DID ≤ 0.5 = 0
10	Ferritin Raised (Fer) [17]	Fer > 322 ng/ml = 1, Fer ≤ 322 ng/ml = 0

**Table 2:** qSOFA variables

Serial Number	Features	Scoring
1	Altered Mental Status	Glasgow Coma Scale < 15 = 1, Glasgow Coma Scale 15 = 0
2	Respiratory rate ≥ 22 per minute	Respiratory Rate ≥ 22 = 1, Respiratory Rate < 22 = 0
3	Systolic Blood Pressure ≤ 100 mmHg	Systolic Blood Pressure ≤ 100 mmHg = 1, Systolic Blood Pressure > 100 mmHg = 0

### Data collection

**Fig. 1:**
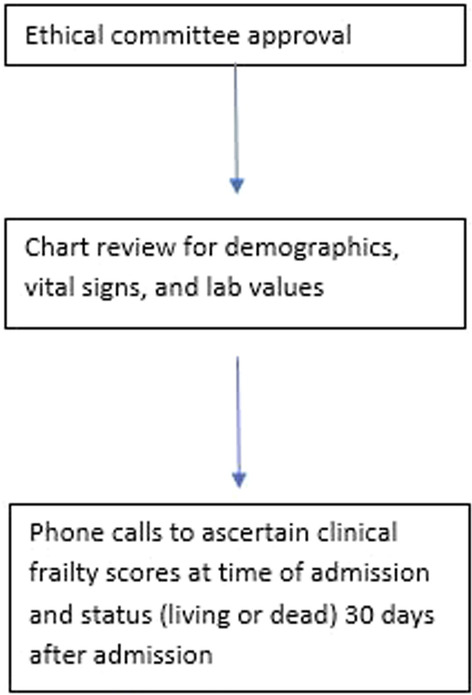
Data collection process

After Ethical committee approval, Study personnel reviewed the medical record to collect the variable data listed in Tables 1 and 2. Although the required sample size was 145,309 patients’ data was used to compensate for any missing data. To ascertain clinical frailty scores at the time of admission, phone calls were made to patients and/or relatives. In cases of no response, attempts to contact them were made two more times. This is summarized in Fig. 1. To maintain good reporting practice, the TRIPOD checklist was used [17]. The data collection process is highlighted in the Fig. 1. Lab values used were those collected on initial presentation to the emergency department.

### Statistical analysis

Data was entered into Microsoft Excel 2010. Data imputation and management was performed using R-software (Version 1.4.1). Data was entered and analyzed using SPSS version-21 (IBM Corp. Released 2012. IBM SPSS Statistics for Windows, Version 21.0. Armonk, NY: IBM Corp). Shapiro–Wilk test was applied to check the hypothesis of normality of all quantitative variables. The qSOFA, CoSAS and in-hospital mortality were our units of analysis. Categorical variables were reported as frequencies and proportions. Descriptive data was reported as mean with standard deviation. The median along with Inter-quartile range was reported for non-normally distributed quantitative data. Receiver operating characteristic analysis (ROC) for in-hospital mortality was performed and area under the curve (AUC) along with its 95% CI was obtained as prognostic strength by comparing utility of COVID-19 Severity Assessment Score (CoSAS) and qSOFA. The identification of cut-off points was done using Youden’s index of receiver operator characteristic (ROC) curve. Optimal cut-off values were chosen to maximize the sum of sensitivity (Se) and specificity (Sp). Positive predictive values (PPV), negative predictive values (NPV), positive likelihood ratios (+ LR) and negative likelihood ratios (-LR) were also assessed. Model for logistic regression for significant results was run to assess different factors to predict in-hospital mortality. Two-sided p-value of ≤ 0.05 was taken as criteria of statistical significance.

## Results

The total number of patients included were 309 out of which 204 were males and 105 were females. Male to female ratio was found to be 1:2. Males are more predominant in our study. The mean age of included patients was 58.9 (± 14). Table 3 demonstrates the descriptive and baseline characteristics found in infected patients of COVID-19 for 28 day mortality. Average length of hospital stay was 7 days.

**Table 3:** Descriptive & baseline characteristics in study patients

Age in years (Mean ± SD)	58.9 (± 14)
Length of Hospital Stay (Days) (Median [IQR])	7 [5–11]
Gender	
Male	204 [66%]
Female	105 [34%]
Comorbids	
HTN	167 [54%]
DM	135 [43.7%]
IHD	45 [14.6%]
CKD	30 [9.7%]
Respiratory illness	28 [9.1%]
CVA	7 [2.3%]
Immunocompromised	14 [4.5%]

Table 4 displays study patient laboratory and clinical parameters. Of the 309 patients, data regarding their 28 day mortality was missing for some. This is because our hospital still uses a paper-based filing system and data values are occasionally missing in files. This meant we could not complete the socring for some patients. Therefore, we included 291 patients in the final analysis. The mean (standard deviation) values of biomarkers were NLR = 7.82 (4.6 -12.99), ferritin 813.2 (383.8–1283.9) and D-Dimer of 1.3 (0.7–3.7). With regards to comorbids, it was observed that majority of patients were hypertensive 167 (54%) followed by diabetic 135(43.7%). On initial triage assessment of the patients the median [Interquartile range] of Systolic Blood Pressure (SBP) was 132 [118–146] mmHg, Glasgow Coma Scale (GCS) of 15, Clinical Frailty Score (CFS) of 3 [2, 3] and a respiratory rate of 30 [25–40] breaths/min. Oxygen saturation was 90% [82% to 96%].

**Table 4:** Study patient laboratory and clinical parameters

Emergency triage vitals	
SBP (mmHg)	132 [118–146]
CFS	3 [2, 3]
Respiratory rate (RR)	30 [25–40]
Oxygen Saturation (%)	90 [82–96]
Biomarkers	
Neutrophil Lymphocyte Ratio	7.82 [4.6–12.99]
Ferritin	813.2 [383.8–1283.9]
D-Dimer	1.3 [0.7–3.7]

As seen in Table 5, The mean CoSAS score of patients was 5.5 (± 1.4) ranging between (0–9) while the mean qSOFA Score was 0.8 (± 0.6)range (0–3). 121 (41.6%) of the sample had a low CoSAS score (< 6) with the remainder scoring ≥ 6. For qSOFA, only 32 (12.4%) had a low score with the remainder scoring ≥ 2.

**Table 5:** CoSAS and qSOFA scores in study patients

CoSAS score	5.5 (± 1.4) [0–9]
qSOFA score	0.8 (± 0.6) [0–3]
CoSAS risk score	
Low risk (< 6 score)	121 [41.6%]
High risk (≥ 6 score)	170 [58.4%]
qSOFA risk score	
Low (< 2 score)	227 [87.6%]
High (≥ 2 score)	32 [12.4%]

Table 6 shows the comparison between the CoSAS and qSOFA in predicting length of stay and 28 day mortality of COVID 19 infected patients. Patients with a high CoSAS score were more likely to be male and older (≥ 50 years). They were more likely to be diabetic, hypertensive, have ischemic heart disease and chronic kidney disease. Interestingly, they were more likely to have lower CRP scores on presentation. Most importantly, a high CoSAS score increased chances of 28 day mortality. There was no significant difference in length of hospital stay in both CoSAS groups.

**Table 6:** Comparison CoSAS with qSOFA in accurately predicting 28 day mortality in adult patients with COVID-19 infection among all baseline and clinical characteristics parameters

Study characteristics	CoSAS Score		p-value	qSofa Score		p-value
	Low (< 6 score)	High (≥ 6 score)		Low (< 2 score)	High (≥ 2 score)	
N	121	170	-	260	31	–
Gender						
Male	61 [50%]	131 [77%]	< 0.001*	174 [67%]	18 [58%]	0.325
Female	60 [50%]	39 [23%]		86 [33%]	13 [42%]	
Mortality						
Alive	117 [97%]	110 [65%]	< 0.001*	220 [85%]	7 [23%]	< 0.001*
Expired	4 [3%]	60 [35%]		40 [15%]	24 [77%]	
Cormorbidities						
Hypertension (HTN)	54 [45%]	105 [62%]	0.004*	141 [54%]	18 [58%]	0.685
Diabetes (DM)	39 [32%]	94 [55%]	< 0.001*	116 [45%]	17 [55%]	0.280
Ischemic Heart Disease (IHD)	9 [7%]	35 [21%]	0.002*	38 [15%]	6 [19%]	0.486
Chronic Kidney Disease (CKD)	6 [5%]	24 [14%]	0.011*	26 [10%]	4 [13%]	0.615
Respiratory Illness	9 [7%]	16 [9%]	0.554	24 [9%]	1 [3%]	0.259
Cerebrovascular accident (CVA)	1 [1%]	6 [4%]	0.138	4 [2%]	3 [10%]	0.005*
Immunocompromised	3 [3%]	11 [7%]	0.117	13 [5%]	1 [3%]	0.663
Age in years	58.04 [49.65–66.62]	72.83 [55.8–79.78]	< 0.001*	50.79 [43.03–65.05]	64.53 [55.78–72.29]	< 0.001*
Length of Hospital Stay (Days)	7 [5–12]	7 [4–11]	0.601	7 [4–13]	7 [5–11]	0.985
GCS	15 [15–15]	13 [11–14]	< 0.001*	15 [15–15]	15 [15–15]	0.885
CFS	2 [2, 3]	3 [2–6]	0.080	2 [2, 3]	3 [2, 3]	0.004*
SBP	132 [118–147]	134 [100–150]	0.311	132 [117–142]	132 [118–150]	0.875
RR	32 [25–40]	28 [24–40]	0.807	30 [24–40]	32 [26–40]	0.619
Saturations	90 [80–96]	88 [84–98]	0.446	88 [78–96]	91 [83–96]	0.242
Neutrophil Lymphocyte Ratio (NLR)	7.99 [4.97–13.35]	7.89 [5.39–14.47]	0.797	6.2 [3.03–10.09]	9.84 [6.15–15]	< 0.001*
C-reactive Protein (CRP)	146.06 [67.53–197.87]	131 [29.69–160.68]	0.048*	143.91 [54.53–188.45]	142.71 [71.36–199.58]	0.074
Ferritin	815.9 [397.5–1258.5]	924.8 [292.3–1614.5]	0.497	627.6 [235.1–1108.7]	904.25 [467.7–1360.2]	0.001*
D-dimer	1.3 [0.7–3.9]	2.2 [1.1–4.5]	0.029	0.8 [0.5–1.6]	1.9 [1.1–4.6]	< 0.001*

*Significant at 5%

A high qSOFA score was associated with higher mortality, age, clinical frailty score, NLR, Ferritin and D-dimer levels. Covid-19 patients with histories of CVAs also scored high qSOFAs. qSOFA also does not accurately predict length of hospital stay.

**Fig. 2:**
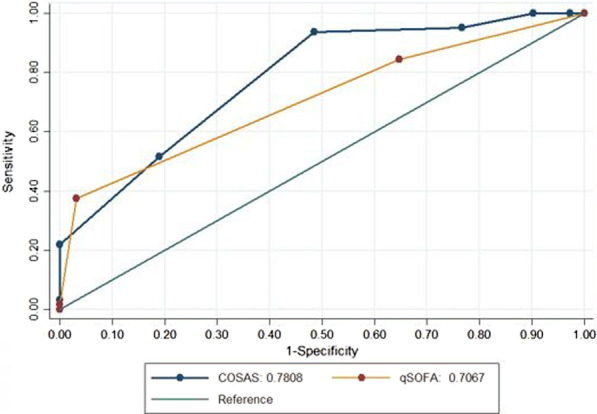
The area under the curve of CoSAS & qSOFA scoring systems for predicting in-hospital mortality

The area under the curve (AUC) of CoSAS & qSOFA scoring systems for predicting in-hospital mortality are presented in Fig. 2. AUC of both scoring criteria system were 78.08% (95% C.I 72.2–83.8%) and 70.6% (63.5–77.5%) respectively. It was observed that there is a statistically significant difference between both scoring criteria system, but CoSAS was found to be reliable and higher as compared to qSOFA (P-value < 0.001*; Fig. 2).

Risk stratification of both scores, established on the main Youden Index cut points, with the best cutoff value >  = 6 of CoSAS was used to predict in-hospital mortality with a sensitivity, specificity, and LR + , of 93.75%, 51.54% and 1.9347 respectively. Similarly, the ROC analysis and diagnostic accuracy analysis of qSOFA score with a cut point >  = 2 was used with a sensitivity, specificity, and LR + , of 84.38%, 35.24% and 1.3029 respectively (See Table 7).

**Table 7:** The Diagnostic accuracy of CoSAS & qSOFA models in calculating in-hospital mortality

	CoSAS Score	qSOFA Score
AUROC	78.08% [95% C.I 72.2–83.8%]	70.6% [63.5–77.5%]
Cut point	(≥ 6)	(≥ 2)
Sensitivity	93.75%	84.38%
Specificity	51.54%	35.24%
LR +	1.9347	1.3029
LR-	0.1213	0.4434

*AUC* area under the curve of the Receiver Operating Characteristic

*95% CI* 95% confidence interval

*CoSAS* Covid-19 Severity Assessment Score

*qSOFA* Quick Sequential Organ Failure Assessment

^⁎^*p* < 0.05

Tables 8 and 9 show mortalities for each score of CoSAS and qSOFA respectively. Mortality progressively increases for a rise in each point in both scores with a large jump at 6 for CoSAS and at 2 for qSOFA.

**Table 8:** Mortality for each point of CoSAS

CoSAS score	Deaths for each score	Total patients
2	0 (0%)	6
3	0 (0%)	16
4	3 (8.8%)	34
5	1 (1.5%)	65
6	27 (28.7%)	94
7	19 (30.6%)	62
8	12 (100%)	12
9	2 (100%)	2
Total	64	291

**Table 9:** Mortality for each point of qSOFA

qSOFA score	Deaths for each score	Total Patients
0	10 (11.1%)	90
1	30 (17.6%)	170
2	23 (76.7%)	30
3	1 (100%)	1
Total	64 (22%)	291

## Discussion

The main findings of our study can be summarized as follows: (1) CoSAS more accurately predicted 28 day mortality in an adult patient with severe covid-19 illness as compared to qSOFA (AUROC 0.78 vs 0.70); (2) Age of more than 55 years, male gender and having previous co-morbidities such as HTN, DM, IHD and CKD are all predictors of severe covid-19 illness. Our study has been conducted in Pakistan at a tertiary care center enrolling more than 200 patients to create an accurate prognostication tool for severe COVID-19 illness. The lack of a specific risk-scoring system for COVID-19 prompted the use of other previously validated screening tools such as qSOFA. Each carrying its innate advantages and disadvantages, CoSAS was hypothesized to be a risk-scoring tool in the evaluation of severely ill COVID-19 patient presenting to the emergency department. Although not validated, the information from a simple screening model may provide useful prognostic information to an Emergency Department and admitting clinicians, thereby potentially directing scarce personnel and medical resources towards those hospitalized individuals who are at the greatest risk of dying.A major strength of our study is that it incorporates clinical parameters that have consistently been shown to be linked with COVID-19 severity [18]. An early large study out of Italy conducted on ICU patients demonstrated a link between COVID-19 severity and age and comorbidities, especially hypertension. Our study validates these findings [19]. Demographically many studies have suggested age > 55 years and the male sex both carry a higher predictive outcome for increased severity [20]. Our study reflected similar results with regards to mean age for low risk (< 6) being 58 years and high risk (≥ 6) associated with > 72 years of age. The male gender has also been a significant predictor of covid-19 illness. The American College of Cardiology along with the CDC have both stated that that male gender carries a higher risk of severe covid (20,21). Fatality rates were highest for cardiovascular disease (10.5%) compared with diabetes (7.3%), COPD (6.3%), hypertension (6.0%), and cancer (5.6%). In contrast, patients without pre-existing conditions had a fatality rate of < 1% [21]. A large analysis of 308,010 COVID-19 adults hospitalized at US academic centers showed that males have a higher rate of respiratory intubation and longer length of hospital stay compared to females and have a higher death rate even when compared across age groups, race/ethnicity, payers, and co-morbidity [22]. Our study also supported these findings as a statistically significant (p < 0.001) result for the male gender was noted to be a predictor for increased severity in covid-19 illness. CoSAS also has statistically significant results for patients that carry HTN (0.004), DM (< 0.001), IHD (0.002) and CKD (0.011), respectively. A limitation in the demographic variables of our study is weight-based categorization. Obesity has been well-documented as a variable that causes increased risk of severe covid related illness [23]. This is likely due to the emergency department unable to document weight during high-risk patient resuscitative procedures associated with large volumes and diminished resources.

qSOFA, consisting of three clinical variables (mental status, respiratory rate, and blood pressure), has been proposed as a rapid screening tool for infected patients [24]. Some studies have concluded that qSOFA score and severity of covid illness have a positive correlation [25]. Whereas others have negated this notion stating a score that is based on altered mentation and circulatory collapse is not created to accurately predict mortality in a virus that leads to ARDS [26, 27]. Our screening tool utilized a Prognostic Multivariable Modelling Design based on data readily available in the first 24 h of hospitalization to predict in-hospital mortality of COVID-19 patients. It was proven with stringent data analysis that CoSAS has a superior prognostic accuracy to qSOFA (shown in Table 7) as proven by the ROC of CoSAS vs qSOFA as 0.78 vs 0.706, respectively. This stands true as CoSAS incorporated much more variables providing a statistically superior result as compared to qSOFA. qSOFA risk stratification scoring accurately predicted an association with age, CFS, NLR and D-Dimer levels whereas both CoSAS and qSOFA were unable to accurately predict length of stay (0.0601 vs 0.985 at CI of 95). Similarly, our study also confirmed that advanced age, male gender, elevated levels of CRP, and previous comorbidities were predictive of in-hospital mortality as was stated in other analyses [23, 28–30]. D-dimer levels obtained on admission accurately predicted mortality which was seen in the CoSAS and qSOFA models. Although CoSAS takes into account 10 factors of any patient on arrival, the study was unable to find a statistically significant relation with CoSAS score and CFS (0.08), Oxygen Saturation at presentation (0.446), systolic blood pressure (0.31), NLR (0.79) or Ferritin (0.49). qSOFA although only requiring 4 initial values showed a statistical significance with CFS (0.004), NLR (< 0.001), Ferritin (0.001) and D-Dimer levels (< 0.001).

CoSAS (high score ≥ 6) for predicting 28 day mortality included: age, gender, clinical frailty score, oxygen saturation, co morbidities, systolic blood pressure, NLR, CRP, DID and ferritin showed an AUC of 0.78 with a sensitivity of 0.93 and specificity of 0.51. CALL score (high risk > 10) for predicting clinical progression of Covid-19 illness included: co-morbidities, age, lymphocyte count, and lactate dehydrogenase was shown to have an AUC of 0.91 with a sensitivity of 0.45 and specificity of 0.97 [31]. NOCOS calculator (high risk: > 51.6%) for predicting 7 day survival included: serum blood urea nitrogen, age, absolute neutrophil count, red cell distribution width, oxygen saturation, and serum sodium and was shown to have an AUC of 0.82 with a sensitivity of 0.89 and specificity of 0.54 [32]. qCSI score (high risk > 4) for respiratory failure within 24 h included: respiratory rate, minimum recorded pulse oximetry, and nasal cannula flow rate requirement was found to have an AUC of 0.81 with a sensitivity and specificity of 0.79 [33]. 4C mortality score (high risk > 9) for predicting in-hospital mortality included: age, sex, number of comorbidities, respiratory rate, pulse oximetry on room air, Glasgow coma scale, serum urea, and C-reactive protein showed an AUC of 0.78 with a sensitivity of 0.93 and specificity of 0.41 [5].

While this score performs similarly and in some cases inferiorly to other well-known scores, it has value in that it was specifically designed to be used at the point of first contact in the emergency department, allowing one to triage those who would benefit from care. It is also the first such study to be carried out in the Pakistani population. This study also shows that qSOFA is inferior to CoSAS in the COVID-19 patient. It also reaffirms what we know about qSOFA’s performance as a COVID-19 severity prediction tool [11] Family members would also hesitate to take life-changing decisions on behalf of their loved ones based off a score that can be calculated the minute they arrive in the emergency department. They would be more amenable to make informed decisions if they are presented with evidence after initial resuscitation in the emergency department. Another important reason to develop a score that can be used in the emergency department is that during the pandemic when hospitals run out of beds to admit new patients, emergency department boarding takes place where COVID-19 patients may be stuck in the emergency department for upto a few days. Therefore an triaging tool at this time becomes all the more important to create space for those who will most likely benefit from care.

There are certain findings in our results that seem to be counterintuitive. For example, presenting vitals and lab values tend to be similar in both high and low risk groups in both scores. The major drivers of severity are age, gender and comorbidities as has been shown in previous studies. Our study also shows that lab values on their own should not be used to predict mortality or severity. Having said that, our study only included the sicker patients who required admission to a non-general ward. Perhaps those in the general ward would have lower lab values.

There are certain limitations to our study that have been identified. As it was a single tertiary care center study in Karachi, Pakistan we were not able to demonstrate whether race or ethnicity affected outcomes. The study was limited to the emergency department and was thus unable to follow up with these patients nor was there any inclusive variable of whether any these patients required Non-Invasive Mechanical Ventilation (NIMV) or Mechanical Ventilation. The sample size was also much smaller than those in other studies. Due to incomplete files, there was also some missing data. Attempts to counteract this limitation included increasing our sample size beyond the minimum 146. While our study shows that CoSAS performs better than qSOFA in predicting mortality, an advantage qSOFA still holds is that it can be calculated within minutes, whereas CoSAS requires lab results which may not be available for hours. Another limitation is that our patient data is from the pre-vaccination era. It is not known how a vaccinated population would score on CoSAS. As our hospital uses a hybrid paper/electronic health record system, many parameters that would make our study more robust are missing, such as reason for ICU admission and percent requiring mechanical ventilation. This is a major limitation of our study and further research in this direction would undoubtedly add value and further refine CoSAS.

## Conclusions

CoSAS is an accurate score to predict Covid-19 mortality in the Pakistani population. CoSAS could predict prognosis very early in patient care through risk stratification and deciding ceiling of care and subsequently help in managing hospital resources. Further, it can assist in leveraging limited supplies of medications, ventilators and ICU beds which is a major concern in low resource settings. It performs better when compared to compared to qSOFA in severe and critical Covid-19 patients. Further studies are needed to externally validate this score.

## Data Availability

The datasets used and/or analyzed during the current study are available from the corresponding author on reasonable request.
